# Maternal infection with SARS‐CoV‐2 during early pregnancy induces hypoxia at the maternal–fetal interface

**DOI:** 10.1111/cpr.13749

**Published:** 2024-10-07

**Authors:** Xiaohui Shi, Chenxiang Xi, Baoxing Dong, Zihui Yan, Wenqiang Liu, Shaorong Gao, Di Chen

**Affiliations:** ^1^ Center for Reproductive Medicine of The Second Affiliated Hospital, Center for Regeneration and Cell Therapy of Zhejiang University‐University of Edinburgh Institute (ZJU‐UoE Institute), Zhejiang University School of Medicine Zhejiang University Hangzhou Zhejiang China; ^2^ Shanghai Key Laboratory of Maternal Fetal Medicine, Clinical and Translational Research Center of Shanghai First Maternity and Infant Hospital, Shanghai Institute of Maternal‐Fetal Medicine and Gynecologic Oncology, Frontier Science Center for Stem Cell Research, School of Life Sciences and Technology Tongji University Shanghai China; ^3^ Dr. Li Dak Sum and Yip Yio Chin Center for Stem Cell and Regenerative Medicine Zhejiang University Hangzhou China; ^4^ State Key Laboratory of Biobased Transportation Fuel Technology Zhejiang University Haining Zhejiang China

## Abstract

The coronavirus disease 2019 (COVID‐19) pandemic increases the risk of adverse fetal outcomes during pregnancy. Maternal infection during pregnancy, particularly with cytomegalovirus (CMV), hepatitis B and C virus, and human immunodeficiency virus can have detrimental effects on both mother and fetus, potentially leading to adverse outcomes such as spontaneous abortion or neonatal infection. However, the impact of severe acute respiratory syndrome coronavirus (SARS‐CoV‐2) infection on the maternal–fetal interface remains poorly understood. In this study, we initially utilised immunofluorescence and immunohistochemical to investigate placental samples from pregnant women who were infected with SARS‐CoV‐2 during the first trimester. Our data indicate that infection in the first trimester induces an upregulation of hypoxia inducible factor (HIF) levels at the maternal–fetal interface. Subsequently, single‐cell RNA sequencing and metabolomics sequencing analyses reveal alterations in maternal–fetal interface. Remarkably, immune cells exhibited low expression levels of HIF possibly associated with immune activation. Furthermore, our findings demonstrate a gradual reduction in transcriptome and metabolic changes as gestation progressed beyond 12–16 weeks compared to samples obtained at 6–8 weeks gestation. Overall, our study suggests that early‐stage SARS‐CoV‐2 infection during the first trimester leads to severe hypoxia and aberrant cell metabolism at the maternal–fetal interface which gradually resolves as pregnancy progresses. Nevertheless, these abnormal changes may have long‐term implications for maternal–fetal interface development.

## Graphical abstract

Severe acute respiratory syndrome coronavirus (SARS‐CoV‐2) infection can cause hypoxia at the maternal–fetal interface in the first trimester, resulting in differential gene expression in different cells and metabolic abnormalities, which are weakened but persist in the second trimester.

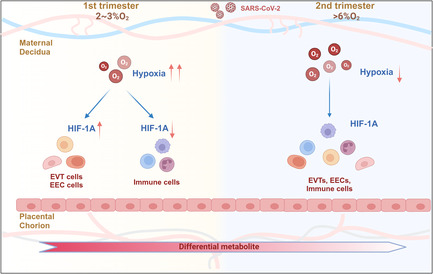

## 1 INTRODUCTION

The coronavirus disease 2019 (COVID‐19) pandemic caused by the severe acute respiratory syndrome coronavirus (SARS‐CoV‐2) has created an unprecedented global health crisis.^1^ Current research on COVID‐19 remains insufficient, particularly regarding its impact on pregnancy. Notably, a substantial amount of data now exists that reveals how infection can affect pregnancy outcomes. Pregnant individuals with COVID‐19 face significantly increased risks of developing hypertensive disorders during pregnancy,^2^ while severe cases have been associated with gestational diabetes.^3^ Potential causes for fetal and neonatal death in pregnancies affected by SARS‐CoV‐2 include severe maternal illness, preterm labour resulting from intrauterine infection and placental insufficiency due to damage.^4^

The interface between the mother and fetus during pregnancy is composed of the endometrium and extra embryonic tissue, including decidua basalis, decidua parietalis and chorion villi. These structures mainly consist of trophoblast cells, and immune cells.^5^, ^6^, ^7^ The placenta serves as a unique organ that establishes a connection between the developing fetus and the uterine wall. It plays crucial roles in providing essential support functions such as nutrient uptake and oxygen exchange throughout gestation for fetal development.^8^ During the initial 8 weeks of human pregnancy, there is a decrease in oxygen levels at the site where the embryo attaches, and during the formation of the placenta due to trophoblasts blocking uterine spiral arterioles,^9^ a lack of oxygen in the environment stimulates the growth of cytotrophoblasts (CTBs) while inhibiting their differentiation along the invasive pathway.^10^, ^11^, ^12^ However, once hemochorial placentation is established, there is an increase in oxygenated blood flow to the uterus, resulting in higher oxygen levels within the placenta. The concentration of oxygen plays a significant role in regulating both proliferation and invasion of CTBs in cultured explants of early gestation human placentas.^13^ Insufficient invasion of trophoblasts and impaired remodelling of uterine spiral arteries during this period can lead to increased blood flow, resulting in damage to villous architecture and an elevated risk of spontaneous vasoconstriction and ischemia reperfusion injury, ultimately causing oxidative stress.^14^ Abnormal placental invasion, compromised development, and functional impairment have been linked to various pregnancy complications, including unexplained abortion,^15^ preeclampsia, fetal growth restriction,^16^ placental abruption^17^ and premature birth.^18^ Hypoxia signalling is a characteristic feature of COVID‐19 pandemic, indicating its potential association with the occurrence and progression of SARS‐CoV‐2 infection.^19^ Hypoxia inducible factor (HIF) transcription factors are central to cellular response under hypoxic conditions as they sense low oxygen levels within cells, leading to metabolic changes, regulation of cell growth, control over inflammatory responses and other functions.^20^, ^21^ In dynamic environment of pregnancy, HIF appears to play a pivotal role in placental development and function as important mediators.^13^

In this study, we aim to investigate the potential impact of COVID‐19 pandemic on oxygen levels at the maternal–fetal interface. Additionally, using single‐cell RNA sequencing (scRNA‐seq) and metabolomics sequencing technology to comprehensively assess its effects and explore the persistence of these changes caused by COVID‐19 pandemic over an extended period. Ultimately, our study reveals a heightened hypoxic state at the maternal–fetal interface during the early stage of SARS‐CoV‐2 infection, providing valuable insights into potential adverse effects on pregnant women.

## 2 MATERIALS AND METHODS

### 2.1 Experimental models and subject details tissue collection

Human chorion, decidua and placenta samples were obtained immediately within minutes of delivery at Shanghai First Maternity and Infant Hospital. Informed consent was obtained from all enrolled subjects. All participants in this study were healthy and did not report any significant comorbidities or complications with pregnancy. The pregnant women were infected with SARS‐CoV‐2 during their 1–3 weeks of pregnancy. There were 10 cases in the SARS‐CoV‐2 infection group and 5 cases in the control group, with gestational ages of 6–8 weeks (first trimester), and 11 cases in the COVID‐19 group and 8 cases in the control group, with gestational ages of 12–16 weeks (second trimester). The maternal clinical information is shown in Tables S1 and S2. Placentas, including the chorion and decidua basalis (maternal membrane), were obtained from pregnant women without any illnesses. The collected tissues were placed in phosphate‐buffered saline (PBS) containing 0.04% bovine serum albumin (BSA) (SIGMA, A1470) and stored at 4°C for transport.

### 2.2 Mechanical dissociation and enzymatic digestion

To remove any excess blood, the tissue was washed by immersing in 15 mL of PBS in a 10 cm Petri dish. Following this, we accurately weighed 2–3 g of the tissue using an analytical balance and transferred it into the prepared dissociation solution. The dissociation solution consisted of DMEM/F12 (Gibco, 11320‐033) containing 0.1% collagenase A (Sigma, 10103586001). Next, we minced the tissue into small pieces within the dissociation solution using a pair of sharp dissecting scissors. These minced samples were then incubated in a water bath, preheated to 37°C, for a duration of 30 minutes. To ensure complete digestion, gentle inversion of the samples was performed every 10 minutes during the incubation period.

### 2.3 Cell filtration and erythrocyte lysis

Following tissue dissociation, the resulting cell suspension was filtered through a 100 μm cell strainer (Biofil, CSS013100) into a 50 mL centrifuge tube. The tube was then centrifuged at 1000 rpm for 5 minutes at 4°C. Carefully, the supernatant was aspirated without disturbing the pellet. Next, the pellet was resuspended in 5 mL of Red Cell Lysis Buffer (TIANGEN, RT122‐02), and the cell suspension was incubated at room temperature for 3–5 minutes with gentle tapping every minute to prevent settling during lysis. To stop the reaction, 0.04% BSA solution was added to a final volume of 50 mL in a centrifuge tube. Once again, the tube was centrifuged at 1000 rpm for 5 minutes at 4°C. The supernatant was then aspirated without disturbing the pellet, which was gently resuspended in 1 mL of 0.04% BSA solution. Finally, the cell suspension was filtered through a 40 μm cell strainer (BIOFIL, CSS01340) into a 15 mL centrifuge tube.

### 2.4 Western blot

The tissue was lysed using radio immunoprecipitation assay lysis buffer buffer (Beyotime, P0013C) following the manufacturer's protocol for protein analysis. Subsequently, the resulting protein lysates were mixed with Omni‐Easy™ Protein Sample Loading Buffer (Denaturing, Reducing, 5×) (Epizyme, LT101s) to prepare for western blotting analysis. For gel electrophoresis, the proteins were heat‐denatured at 100°C for 20 minutes and loaded onto an 8% sodium dodecyl sulfate polyacrylamide gel electrophoresis gel along with a PageRuler Prestained Protein Ladder (Thermo, 26616). A constant voltage of 80 V was applied for the stacking gel, followed by 100 V for the running gel. Next, the proteins were transferred to a polyvinylidene fluoride membrane (Millipore, IPVH00010) at a constant electric current of 300 mA for 120 minutes. To block non‐specific binding, the membrane was incubated in 5% non‐fat milk for 90 minutes. Following this step, primary antibodies Rabbit‐anti‐human‐HIF‐1 alpha (1:1000 dilution, Abcam, ab51608) and Mouse‐anti‐human‐B‐actin (1:2000 dilution, HuaBio, EM21002) were added and incubated overnight at 4°C. On the second day, after washing three times with tris‐buffered saline containing 0.1% Tween20 (tris buffered saline with tween [TBST]), horseradish peroxidase (HRP)‐linked‐anti‐rabbit IgG (1:2000 dilution, CST, 7074P2) and HRP‐linked anti‐mouse IgG (1:2000 dilution, CST, 7076P2) secondary antibodies were added and incubated for 2 h at room temperature. After another three washes with TBST, used enhanced chemiluminescence reagent (ECL) (advansta, K‐12045‐D50) to visualise the membrane by LI‐COR Odyssey Fc Imaging System (LICORbio, USA), and used ImageJ to analyse.

### 2.5 RNA extraction and RT‐qPCR

The mRNA expression was quantified using reverse transcription–quantitative real‐time PCR (RT‐qPCR). Total RNA was isolated from tissue samples (chorion, decidua and placenta) using AG RNAex Pro Reagent (Accurate Biology, AG21101). Subsequently, the purity and concentration of the extracted RNA were assessed. Next, complementary DNA (cDNA) synthesis was performed by reverse transcribing the RNA with Evo M‐MLV RT Premix (AG11706). Target cDNA amplification was measured using TB Green® Premix Ex Taq™ II (Tli RNaseH Plus) (Takara, RR420A) for human *HIF‐1A*, Vascular Endothelial Growth Factor A (*VEGFA*), Fms related Receptor Tyrosine kinase 1 (*FLT1*), BCL2 Interacting Protein 3 (*BNIP3*), Mitogen‐Activated Protein Kinase 6 (*MAPK6*) and Pyruvate Dehydrogenase Kinase1 (*PDK1*). RT‐qPCR amplification was conducted on a LightCycler480 System (Roche Diagnostics, USA). To normalise mRNA expression levels, glyceraldehyde‐3‐phosphate dehydrogenase (GAPDH) expression served as an internal control. The fold change in target mRNA expression relative to GAPDH was calculated using the Computed Tomography (CT) (2 − ΔΔCT) method.

### 2.6 Immunofluorescence

For immunofluorescence (IF) staining, the CD45 antibody (Servicebio, GB14038) and HIF‐1 alpha antibody (Abcam, ab51608) were utilised. Initially, paraffin sections were subjected to dewaxing for enhanced antigen retrieval. Subsequently, the regions of interest in the tissue were delineated using an immunohistochemistry (IHC) pen prior to a 30‐min blocking step. Following blocking, primary antibodies were applied via the drop method and slides were incubated overnight at 4°C in a wet box. Post‐incubation, slides were washed thrice with PBS (pH 7.4), followed by addition of secondary antibody (Servicebio, GB25301/GB25303). Incubation at room temperature in darkness was continued for 50 min. Subsequently, slides underwent washing and nuclei counterstaining with 4',6‐diamidino‐2‐phenylindole solution. To minimise tissue autofluorescence, an anti‐fluorescence quenching sealing agent was employed before finally sealing the slides with a coverslip.

### 2.7 Immunohistochemistry

For immunohistochemistry (IHC), tissue samples were fixed in 10% formalin buffer for 6 h at room temperature. Following fixation, the samples were embedded in paraffin. After routine rehydration and antigen retrieval, 5 μm paraffin sections were prepared. These sections were then stained with the rabbit‐anti‐human‐HIF‐1 alpha (Abcam, ab51608). To visualise the staining, the sections were incubated with HRP‐conjugated secondary antibodies and treated with a 3,3’‐diaminobenzidine solution (MXB, KIT‐5920) containing 0.03% H_2_O_2_. The sections were then mounted and counterstained with haematoxylin.

### 2.8 Single‐cell RNA sequencing

All the raw data of scRNA‐seq from our published articles.^22^ DNBelab C Series Single‐Cell Library Prep Set (MGI) was utilised as previously described^23^ for single‐cell RNA‐seq library preparation. In brief, single‐cell suspensions were converted to barcoded scRNA‐seq libraries through steps including droplet encapsulation, emulsion breakage, mRNA captured bead collection, reverse transcription, cDNA amplification and purification. Indexed sequencing libraries were constructed according to the manufacturer's protocol. Concentrations were measured with a Qubit ssDNA Assay Kit (Thermo Fisher Scientific, Q10212), and the libraries were sequenced using a DNBSEQ‐T7 sequencer with the following sequencing strategy: 30‐bp read length for read 1 and 100‐bp read length for read 2.

### 2.9 scRNA‐seq analysis

To identify differentially expressed genes (DEGs) between the control and COVID‐19 groups, the ‘FindMarkers’ function from the ‘Seurat’ package was employed. Genes with a *p* value less than 0.05 and a log2(fold change) greater than 0.5 were considered DEGs. The upregulated and downregulated genes were separately analysed using Metascape to perform GO analysis by following the provided instructions.^24^ For the cytokine score, the ‘AddModuleScore’ function from the ‘Seurat’ package was applied, and the gene set was downloaded from Molecular Signatures Database.^25^ To infer cell–cell communication between different cell types, the R package ‘CellChat’ was utilised by following the standard pipeline.^26^

### 2.10 Metabolomics analysis

All the raw data of metabonomics sequencing from our published article.^22^ Metabolomics analysis was conducted by Shanghai Applied Protein Technology Co., Ltd. Briefly, metabolites from samples were extracted through lysis, sonication and centrifugation. Untargeted metabolomics of polar metabolites was performed, and extracts were analysed using a quadrupole time‐of‐flight mass spectrometer (Sciex TripleTOF 6600) coupled to hydrophilic interaction chromatography via electrospray ionisation. The mass spectrometer was operated in both negative ion and positive ion modes.

The raw mass spectrometry (MS) data were converted to MzXML files using ProteoWizard MSConvert before data processing using XCMS software. In the extracted ion features, only variables having more than 50% of the nonzero measurement values in at least one group were retained. After normalisation to total peak intensity, differentially expressed metabolite analysis was performed, and metabolites with log2 (fold change) >1 and *p* value <0.05 were considered differentially expressed metabolites. Differentially expressed metabolites were then uploaded to MetaboAnalyst 5.0 for kyoto encyclopedia of genes and genomes (KEGG) pathway annotation. Pathways with *p* values <0.05 were considered significantly changed pathways.

## 3 RESULTS

### 3.1 COVID‐19 infection in pregnant women of early pregnancy induces hypoxia at the maternal–fetal interface

**FIGURE 1:**
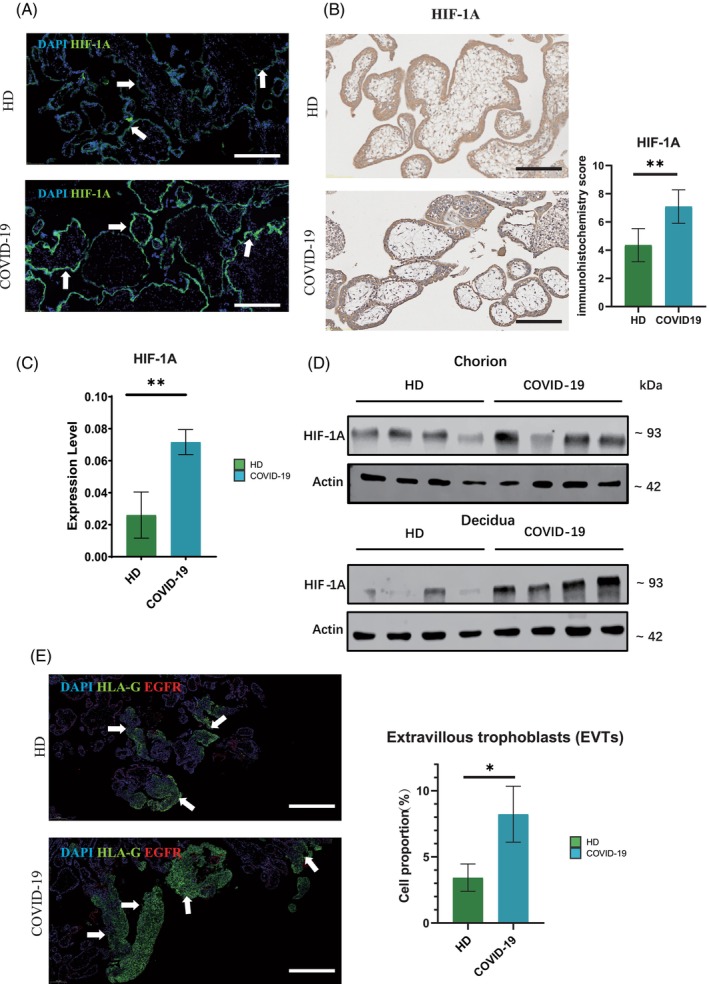
Coronavirus disease 2019 (COVID‐19) infection in pregnant women of early pregnancy induces hypoxia at the maternal–fetal interface. (A) Immunofluorescence staining of Hypoxia Inducible Factor 1 Subunit Alpha (HIF‐1A) in the placental chorion between the 6–8 weeks Healthy Donors (HD) and COVID‐19 groups. Scale bar = 100 μm. (B) Immunohistochemistry of HIF‐1A in placental chorion tissues of the 6–8 weeks HD and COVID‐19 groups. Scale bar = 100 μm. The bar plot showing the immunohistochemistry score of HIF‐1A between the 6–8 weeks placental chorion HD and COVID‐19 groups. **represents *p* < 0.01. (C) RT‐qPCR showing relative expression level of *HIF‐1A* in placental chorion of the 6–8 weeks HD and COVID‐19 groups. ** represents *p* < 0.01. (D) Western blot results show the expression of β‐actin and HIF‐1A in 6–8 weeks placental chorion and maternal decidua in HD and COVID‐19 groups. β‐actin: 42 kDa. HIF‐1A: 93 kDa. (E) Immunofluorescence staining of Epidermal Growth Factor Receptor (EGFR) and Human leukocyte antigen‐G (HLA‐G) in 6–8 weeks placental chorion extravillous trophoblasts (EVTs) of HD and COVID‐19 group. Scale bar = 200 μm. The bar plot showing the percentage of EVTs between the 6–8 weeks placental chorion HD and COVID‐19 groups. * represents *p* < 0.05.

The state of hypoxia is a natural and essential element during the growth of an embryo. Nevertheless, this inherent state makes the embryo vulnerable to potential harm if there is any disturbance in the provision of oxygen.^27^ The SARS‐CoV‐2 primarily targets pulmonary tissue, causing interference with gas exchange and ultimately leading to the emergence of acute respiratory distress syndrome (ARDS) and systemic hypoxia. Consequently, this results in diverse levels of hypoxic harm to other organs.^28^ In order to comprehensively evaluate whether SARS‐CoV‐2 infection can cause more severe hypoxia at the maternal–fetal interface, we collected clinical samples of placental chorion and maternal decidua from pregnant women who underwent abortion at 6–8 weeks as the experimental group. These pregnant women were confirmed as COVID‐19 positive by RT‐PCR at 1–3 weeks of gestation. At the same time, we collected placental chorion and maternal decidua samples from healthy pregnant women without SARS‐CoV‐2 infection at the same gestational age as the control group (all women had voluntary normal abortion), in which there was no difference in age between the SARS‐CoV‐2 infected patients and the healthy pregnant women, and they had no other pregnancy diseases. Therefore, in all subsequent analyses, we targeted all pregnant women with SARS‐CoV‐2 infection (referred to as the COVID‐19 group) and compared them with healthy pregnant women (Healthy Donors as the HD group) without SARS‐CoV‐2 infection. IF experiments on the placental chorion samples of the COVID‐19 group and HD group showed that the normal placental development in early pregnancy was in a hypoxic environment. Hypoxia Inducible Factor 1 Subunit Alpha (HIF‐1A) protein was expressed in the HD group while exhibiting a stronger fluorescence signal in the COVID‐19 group. This indicated that the COVID‐19 group induced hypoxia signalling pathway to promote the expression of HIF‐1A (Figure 1A). At the same time, our IHC experiment showed that HIF‐1A in the chorion of the COVID‐19 group obtained a higher score (Figure 1B). Subsequently, we extracted tissue RNA for real‐time PCR (qPCR) detection, and the results showed that *HIF‐1A* was highly expressed at the transcriptional level in the COVID‐19 group (Figure 1C). Western blot proved that the expression of HIF‐1A protein was higher in the COVID‐19 group, and the maternal decidua group is more obvious than the placental chorion group, which may be related to the maternal infection and hypoxia (Figure 1D). Human leukocyte antigen‐G (HLA‐G) is highly expressed in extravillous trophoblasts (EVTs) that invade the decidual of the uterus, and its expression at the maternal–fetal interface is important for maternal tolerance and maintenance of normal pregnancy.^29^ Previous studies have shown that under hypoxia, villous cytotrophoblast cells (VCTs) are more likely to differentiate into EVT cells to maintain pregnancy.^30^ We then used Epidermal Growth Factor Receptor (EGFR) and HLA‐G to trace EVT cells. Notably, IF results showed a significantly higher proportion of EVT cells in the placental chorion of the COVID‐19 group, which was consistent with previous reports (Figure 1E). Taken together, these observations indicate that maternal COVID‐19 infection in the first trimester may cause severe hypoxia at the maternal–fetal interface.

### 3.2 Characteristics of the study population and single‐cell transcriptome profiles of the maternal–fetal interface

**FIGURE 2:**
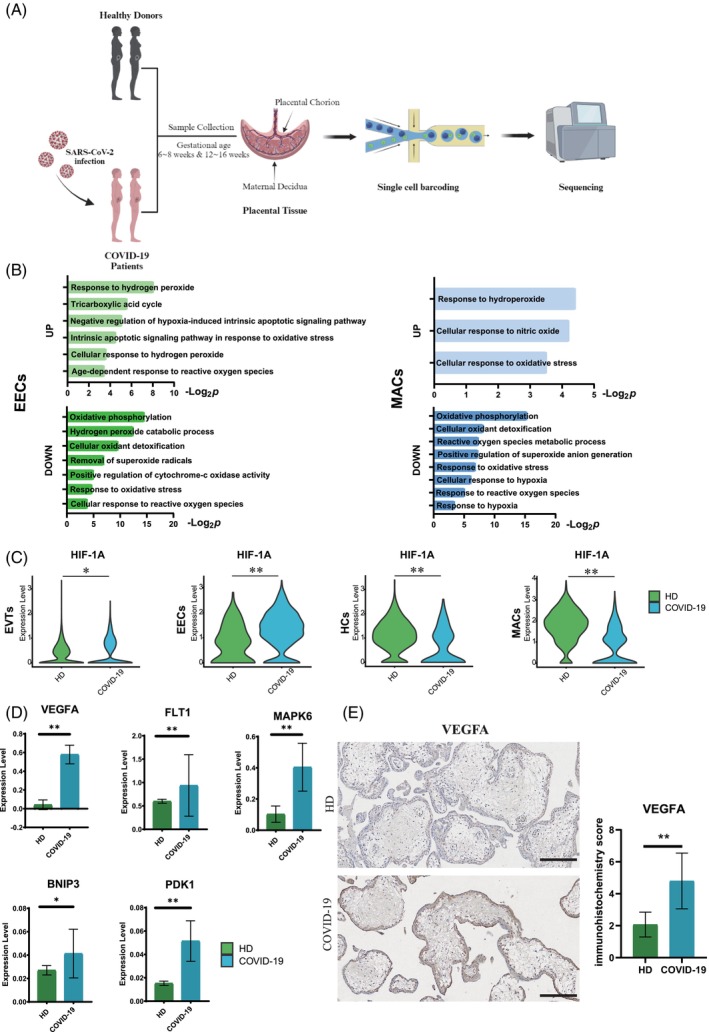
Characteristics of the study population and single‐cell transcriptome profiles of the maternal–fetal interface. (A) Experimental design for the study. 6–8 weeks placental chorion and maternal decidua samples, 12–16 weeks placenta samples were collected from HD and SARS‐CoV‐2 infected pregnancies. The samples were separated and processed by mechanical and enzymatic methods to isolate single cells, which were multiplexed and profiled using single‐cell RNA sequencing. (B) Gene Ontology enrichment analysis of upregulated and downregulated DEGs in Endometrial Epithelial Cells (EECs) and macrophages (MACs) of decidua tissues from pregnant women infected with SARS‐CoV‐2. (C) Violin plots of select DEGs in different cell types of the 6–8 weeks HD and COVID‐19 groups. Expression levels were transformed using logarithm. * represents *p* < 0.05. ** represents *p* < 0.01. (D) RT‐qPCR showing relative expression level of hypoxia‐regulated genes (Vascular Endothelial Growth Factor A [*VEGFA*], Fms Related Receptor Tyrosine Kinase 1 [*FLT1*], Mitogen‐Activated Protein Kinase 6 [*MAPK6*], BCL2 Interacting Protein 3 [*BNIP3*] and Pyruvate Dehydrogenase Kinase1 [*PDK1*]) in placental chorion of the 6–8 weeks HD and COVID‐19 groups. * represents *p* < 0.05. ** represents *p* < 0.01. (E) Immunohistochemistry of VEGFA in placental chorion tissues of the 6–8 weeks HD and COVID‐19 groups. Scale bar = 100 μm. The bar plot showing the immunohistochemistry score of VEGFA between the 6–8 weeks placental chorion HD and COVID‐19 groups. ** represents *p* < 0.01.

Subsequently, we comprehensively examined the impact of SARS‐CoV‐2 infection induced severe hypoxia on the maternal–fetal interface by scRNA‐seq technology (Figure 2A). The collected tissue samples encompassed placental chorion and maternal decidua at 6–8 weeks gestation (All the samples were infected with SARS‐CoV‐2 during the 1–3 weeks of gestation), these tissues were enzymatically dissociated into individual cells.^22^ After eliminating doublets and non‐viable cells, performing dimension reduction and clustering analyses, our investigation successfully identified eight distinct cell clusters within both placental chorion and maternal decidua (Figure S1A,B). Cluster annotations were assigned based on differential marker genes expression analysis. We examined the expression of the marker genes for the corresponding cell types^22^ (Figure S1C). To understand the changes at the maternal–fetal interface after SARS‐CoV‐2 infection in pregnant women, we investigated the gene expression changes in immune cells, including Hofbauer cells (HCs) and macrophages (MACs), as well as Endometrial Epithelial cells (EECs) and EVTs after SARS‐CoV‐2 infection by differential expression analysis. There were significant differences in gene expression levels between COVID‐19 and HD groups (Figure S1D). Through Gene Ontology (GO) term analysis based on differentially expressed genes (DEGs), we observed that genes altered in stromal cells, trophoblast cells and immune cells were associated with cellular responses to hypoxia, oxidative stress response and oxidative phosphorylation (Figures 2B and S2A). This finding suggests that COVID‐19 infection significantly affects oxygen transfer processes at the maternal–fetal interface along with essential mechanisms involved in oxidative phosphorylation for various life processes.

Notably, different cell types exhibited distinct levels of *HIF‐1A* transcription. In EECs and EVTs, the expression level of *HIF‐1A* was higher in the COVID‐19 group compared to the HD group. However, in immune cells such as MACs and HCs, *HIF‐1A* expression was higher in the HD group (Figure 2C). The hypoxic conditions at the early maternal–fetal interface are crucial for preventing maternal immune cells from attacking fetal‐derived semi‐allogeneic cells within the decidua. Therefore, we hypothesised that the lower levels of *HIF‐1A* expression in COVID‐19 group might be associated with immune cells activation. We use CD45 antibodies to identify specific cell types and monitor their activation status.^31^ IF results revealed an increased aggregation of CD45‐positive cells within clusters in the decidua of the COVID‐19 group, suggesting heightened immune cell activation and mobilisation compared to the HD group (Figure S2B).

The *VEGF‐VEGFR* family is particularly important for placental angiogenesis and plays a crucial role in embryonic development, placenta formation, as well as tissue and organ development during pregnancy. Transcriptomic analysis revealed significantly higher expression levels of Vascular Endothelial Growth Factor A (*VEGFA*) and Fms Related Receptor Tyrosine Kinase 1 (*VEGFR1*, *FLT1*) in the COVID‐19 group compared to the HD group, this finding was further supported by qPCR (Figure 2D). Additionally, immunohistochemical results showed a higher score for VEGFA (Figure 2E). Furthermore, qPCR analysis indicated an upregulation of Mitogen‐Activated Protein Kinase 6(*MAPK6*), BCL2 Interacting Protein 3(*BNIP3*), Pyruvate Dehydrogenase Kinase 1(*PDK1*) along with other hypoxia‐regulated genes involved in embryonic development and organ formation (Figure 2D). These findings suggest that severe hypoxia at maternal–fetal interface during early pregnancy leads to differential expression of HIF‐1A across various cells at the maternal–fetal interface. The increased expression of HIF‐1A in EVTs and EECs may be associated with enhanced immune tolerance and cell migration capabilities, while lower levels of HIF‐1A in immune cells may be activated to counteract viral invasion. Moreover, severe hypoxia at maternal–fetal interface during early pregnancy also influences the expression patterns of numerous downregulated genes that are critical for essential biological processes such as embryonic development, cell proliferation and normal pregnancy. Altogether, SARS‐CoV‐2 infection during early pregnancy can lead to severe hypoxia and induce the upregulation of HIF‐1A at maternal–fetal interface. Dysregulated expression of HIF‐1A and its downregulated targets may exert adverse effects on placental and fetal development.

### 3.3 The more severe hypoxia at maternal–fetal interface caused by SARS‐CoV‐2 disappeared with developmental

**FIGURE 3:**
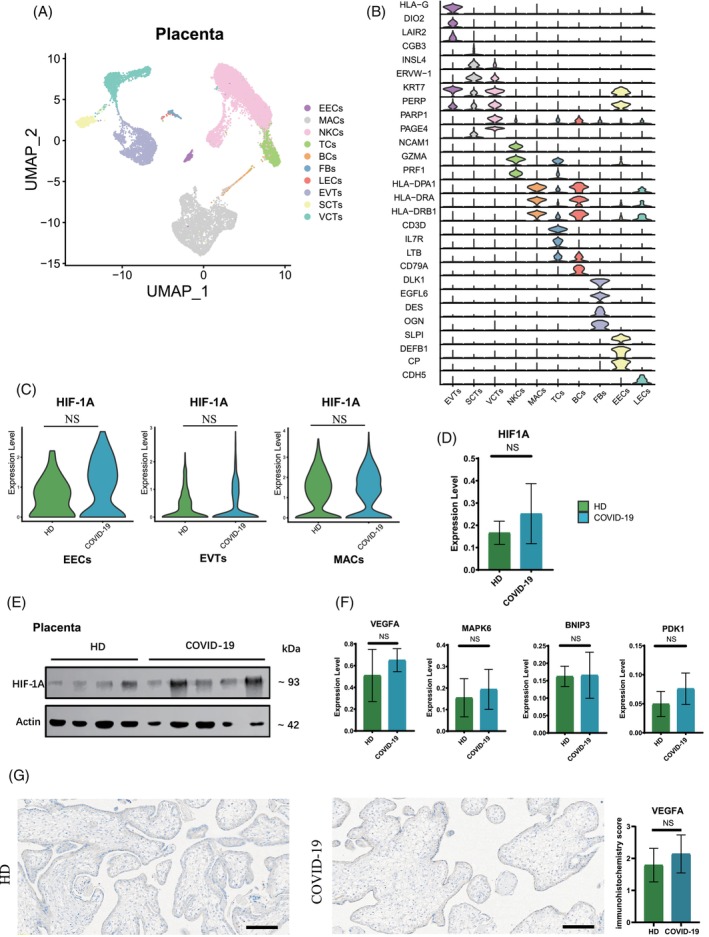
The more severe hypoxia at maternal–fetal interface caused by SARS‐CoV‐2 disappeared with developmental. (A) Uniform manifold approximation and projection plots of 12–16 weeks placenta tissues coloured by cell type. (B) Violin plot of key gene expression markers used for annotations of cells from 12–16 weeks placenta tissues. (C) Violin plots of select DEGs in different cell types of the 12–16 weeks placenta HD and COVID‐19 groups. Expression levels were transformed using logarithm. NS represents no significance. (D) RT‐qPCR showing relative expression level of *HIF‐1A* in placenta of the 12–16 weeks HD and COVID‐19 groups. NS represents no significance. (E) Western blot results show the expression of β‐actin and Hypoxia Inducible Factor 1 subunit Alpha (HIF‐1A) in 12–16 weeks placenta tissues in HD and COVID‐19 groups. β‐actin: 42 kDa. HIF‐1A: 93 kDa. (F) RT‐qPCR showing relative expression level of hypoxia‐regulated genes (Vascular Endothelial Growth Factor A [*VEGFA*], Mitogen‐Activated Protein Kinase 6 [*MAPK6*], BCL2 Interacting Protein 3 [*BNIP3*] and Pyruvate Dehydrogenase Kinase1 [*PDK1*]) in placenta of the 12–16 weeks HD and COVID‐19 groups. NS represents no significance. (G) Immunohistochemistry of VEGFA in placenta tissues of the 12–16 weeks HD and COVID‐19 groups. Scale bar = 100 μm. The bar plot showing the immunohistochemistry score of VEGFA between the 12–16 weeks placenta tissues in HD and COVID‐19 groups. NS represents no significance.

To explore the potential long‐term impacts of hypoxia caused by COVID‐19 on the connection between mother and fetus, we reanalysed scRNA‐seq data of placental samples from pregnant women who tested positive for COVID‐19 using RT‐PCR during 1–3 weeks of pregnancy and chose to terminate their pregnancies at 12–16 weeks as our COVID‐19 group.^22^ Normal placental samples from pregnant women in the HD group at equivalent gestational stages. We have identified ten distinct cell clusters within the COVID‐19 group (Figure 3A). The cluster annotations were determined by examining the expression of marker genes specific to placental cells (Figure 3B). Interestingly, unlike the observations in early samples taken at 6–8 weeks, scRNA‐seq data showed there were no significant differences in *HIF‐1A* expression levels among EECs, EVTs and MACs within 12–16 weeks placental tissues between the COVID‐19 and HD groups at the transcriptional level (Figure 3C). This discovery was further supported by qPCR analysis, which showed similar expression levels of *HIF‐1A* in these tissues (Figure 3D). Furthermore, Western blot demonstrated comparable protein expression levels of HIF‐1A between the HD and COVID‐19 groups (Figure 3E). Moreover, qPCR analysis indicated that inter‐group expression levels of *VEGFA* associated with placental angiogenesis were also similar (Figure 3F), which was confirmed through immunohistochemical experiments (Figure 3G). No significant differences were detected in the expression of other HIF‐1A downregulated genes such as *MAPK6*, *PDK1* and *BNIP3* between the HD and COVID‐19 group (Figure 3F). These findings collectively suggest that there is no substantial increase in HIF‐1A expression at the maternal–fetal interface during 12–16 weeks compared to 6–8 weeks of gestation. This implies that the hypoxia at the maternal–fetal interface may ameliorate with fetal development and progression of pregnancy.

### 3.4 Metabolic effects of hypoxia at the early maternal–fetal interface

**FIGURE 4:**
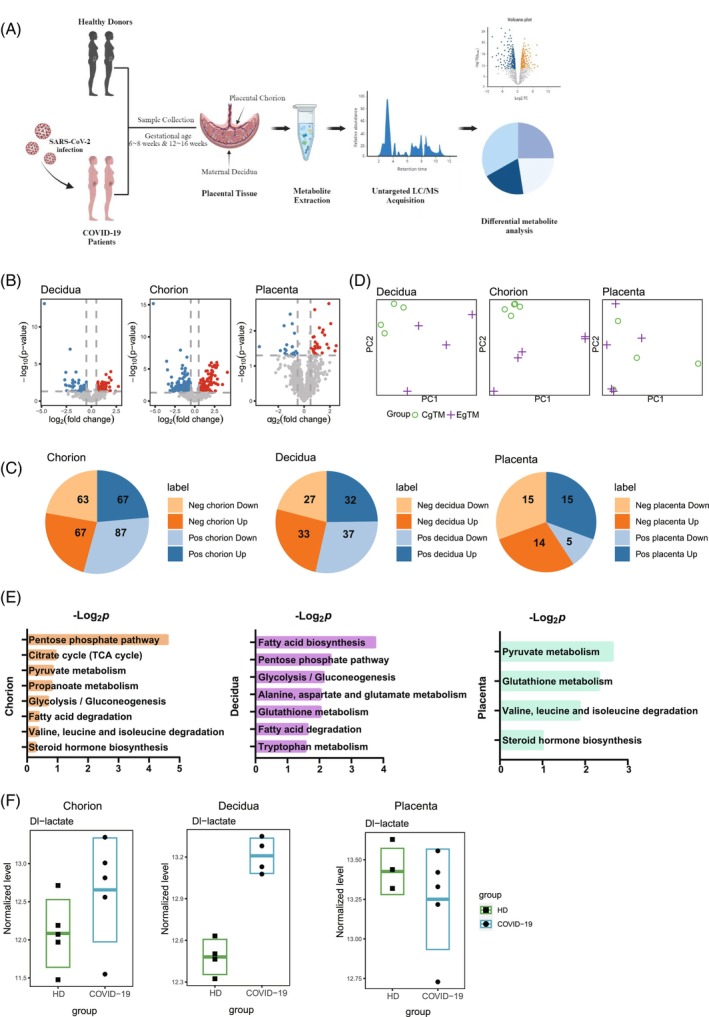
Metabolic effects of hypoxia at the early maternal–fetal interface. (A) Experimental design for untargeted metabolomic analysis. (B) Volcano plot showing the differentially regulated metabolites in placental chorion, maternal decidua and placenta from pregnant women infected with SARS‐CoV‐2. (C) The pie chart showing the counts of differentially regulated metabolites in each group. (D) Principal component analysis (PCA) plots of the samples from three tissues. (E) KEGG analysis of differentially regulated metabolites from three tissues. (F) Box plot showing the DL‐lactate scores of different tissues between the HD and COVID‐19 groups. The mean ± SD of each group is shown in the plot.

It has been discovered that the systemic hypoxic state may have induced a rewiring of metabolism to optimise the overall adaptation of the whole body.^32^ Hypoxia and expression of HIF‐1A transcription factors have been documented to trigger metabolic alterations, with glucose metabolism reprogramming and urea cycle dysfunction identified as underlying pathological mechanisms in COVID‐19.^33^ To explore the impact of severe hypoxia in pregnant women following COVID‐19 infection on cell metabolism, we reanalysed metabolomics sequencing data of placental chorion and maternal decidua at 6–8 weeks, as well as placental samples at 12–16 weeks^22^ (Figure 4A). To compare metabolite regulation between HD and COVID‐19 groups, we performed differential enrichment analysis separately for metabolites enriched in positive ion and negative ion modes (Figure 4B). Our findings revealed a higher number of differentially regulated metabolites in maternal decidua and placental chorion tissues at 6–8 weeks. In contrast, there was a significant decrease in the number of differentially regulated metabolites observed in placental tissues from 12 to 16 weeks (Figure 4C). These results align with our transcriptomic and protein findings, suggesting that COVID‐19 induced hypoxia is more pronounced during the early stages but gradually diminishes along with fetal development.

We conducted a principal component analysis (PCA) of enriched metabolites in tissues at different stages (Figure 4D) and observed that the HD and the COVID‐19 group showed more noticeable variations in tissues between 6–8 weeks, while there were no statistically significant differences observed between groups in tissues from 12–16 weeks. KEGG analysis of differentially enriched metabolites revealed that SARS‐CoV‐2 infection primarily affected placental chorion through alterations in metabolic pathways such as the Pentose phosphate pathway, Citrate cycle (tricarboxylic acid cycle) and Pyruvate metabolism. Maternal decidua, on the other hand, showed changes in Fatty acid biosynthesis and Glutathione metabolism (Figure 4E). Previous studies have demonstrated that these metabolic pathways can be influenced by hypoxia.^34^, ^35^, ^36^, ^37^ Notably, our findings indicated variation in Pyruvate metabolism and Glutathione metabolism within the placenta, suggesting prolonged effects of severe hypoxia caused by COVID‐19 on the maternal–fetal interface. Additionally, under hypoxic conditions DL‐lactate levels rise due to cells impaired glucose oxidation,^33^ we observed a significant elevation in DL‐lactate levels in early stage tissues, especially within maternal decidua (Figure 4F). Prior research has also shown that COVID‐19 infection can lead to increased lactate levels.^38^ In conclusion, our study highlights substantial impacts of COVID‐19 infection on hypoxia related metabolites at the maternal–fetal interface which were partially attenuated by 12–16 weeks of development. However, there remained a small subset of differentially expressed metabolites with potential persistent effects on maternal–fetal development. Taken together, the impact of SARS‐CoV‐2 infection in early pregnancy on the development of maternal–fetal interface may gradually attenuate over time while still persisting.

## 4 DISCUSSION AND CONCLUSION

The impact of COVID‐19 goes beyond the respiratory system, as recent reports and studies have demonstrated significant adverse outcomes in pregnant women, such as increased rates of preterm births (38%), maternal (5%) and neonatal mortality (6%) and vertical transmission (5%) of the virus.^39^ The excessive inflammation caused by SARS‐CoV‐2 infection disrupts gas exchange in the lungs, leading to various complications such as hypoxia, angiogenesis, coagulation and fibrosis in organs like the heart, liver, kidneys and thyroid gland,^40^ which are particularly detrimental in the context of the maternal–fetal interface under the already low oxygen conditions necessary for early pregnancy. The dynamics of SARS‐CoV‐2 transmission further complicate the safety in clinical settings, underscoring the importance of robust infection control measures to shield pregnant women from nosocomial infections.^41^ Additionally, COVID‐19 may negatively affect male fertility, while also causing damage to the central nervous system due to reduced oxygen supply.^42^ There is still uncertainty regarding how COVID‐19 affects maternal–fetal interface under hypoxic conditions, despite its significant implications for human life activities. Through our study, we have demonstrated that infection with COVID‐19 during early pregnancy can result in hypoxia at the maternal–fetal interface. However, further comprehensive research is required to ascertain its potential long‐term impact on fetal development.

Stability of the early intrauterine environment is crucial for embryo implantation and fetal development. In normal pregnancies, a low oxygen environment in the placenta is physiological and necessary during early pregnancy,^13^ as it helps establish a good connection between the fetus and mother while protecting the fetus from maternal immune cell attacks. However, previous studies have shown that abnormal fluctuations in oxygen levels can cause significant damage to the development of the maternal–fetal interface and are associated with common complications in pregnancy.^43^ Therefore, in this study, we collected placental samples from pregnant women infected with SARS‐CoV‐2 during early pregnancy. IF and IHC experiments revealed significantly increased expression of HIF‐1A in the COVID‐19 group. To investigate hypoxic responses in different cell populations at the maternal–fetal interface, we performed scRNA‐seq on 6–8 weeks placental chorion and maternal decidua tissues. The analysis showed elevated HIF‐1A expression levels in cells such as EECs and syncytiotrophoblasts, specifically within the COVID‐19 group. Interestingly, immune cells such as MACs and HCs exhibited decreased HIF‐1A expression compared to the HD group, suggesting a non‐hypoxic state possibly related to immune cell activation. Continuous changes in oxygen concentration may have irreversible effects on fetal development. Therefore, we are concerned about whether the severe hypoxia caused by COVID‐19 will persist at the maternal–fetal interface. Through single‐cell transcriptomic and metabolomic sequencing analyses of placenta at 12–16 weeks of gestation, we found that the state of severe hypoxia gradually alleviates as development progresses. Nevertheless, differential gene expression and metabolites persist, suggesting potential long‐lasting impacts that warrant further investigation.

Common clinical symptoms of COVID‐19 include fever, chills, both dry and productive coughs, lethargy, headache and dyspnea.^44^ Our research reveals a new finding that early pregnancy infections with SARS‐CoV‐2 can induce hypoxia at the maternal–fetal interface. Severe viral infections may affect placental development, leading to spontaneous pregnancy loss or preterm birth.^45^ Given the unique physiological and immune characteristics of pregnant women, placental development and organogenesis in the first trimester of pregnancy make the fetus particularly vulnerable to serious diseases, so the vaccine and pharmacological treatment applications must be approached with caution. In order to reduce the irreversible effects of severe hypoxia induced by COVID‐19 on both mother and fetus, it is recommended to administer adequate oxygen therapy to pregnant patients in the early stages of infection, thereby attenuating viral impact and facilitating maternal–fetal recovery. Challenges in sample collection and research technology limitations have led to few studies on the effects of early pregnancy viral infections on the maternal–fetal interface. The paucity of data on emerging viral infections prevents the development of specialised treatment protocols for early‐stage pregnant women, different from those for the general population. Consequently, this situation escalates health risks for pregnant women, potentially resulting in adverse pregnancy outcomes. Our study provides novel insights into the potential impact of viral infection on the maternal–fetal interface during pregnancy, also supplies clues for the clinical prevention and treatment of viral infection in early pregnancy. According to our research, early‐stage pregnant women can choose customised treatment strategies to address new waves of viral infections and infectious diseases, which helps mitigate the adverse effects on the development of the maternal–fetal interface.

## AUTHOR CONTRIBUTIONS

WL, SG and DC conceived of the project and provided mentoring. XS, CX, BD and ZY designed and performed the experiments. ZY provided bioinformatics support. XS, CX, BD, ZY, WL, SG and DC wrote the manuscript.

## FUNDING INFORMATION

This work was supported by the: National Natural Science Foundation of China awarded to DC (Grant No. 32270835), Zhejiang Natural Science Foundation awarded to DC (Grant No. Z22C129553), Dr. Li Dak Sum & Yip Yio Chin Development Fund for Regenerative Medicine, Zhejiang University, awarded to DC.

## CONFLICT OF INTEREST STATEMENT

The authors declare that they have no conflicts of interest.

## Supporting information


**Data S1.** Supporting Information.

**Data S2.** Supporting Information.

## Data Availability

All data associated with this study are presented in the paper or the Supporting Information. The sequencing data used in this article are available in ArrayExpress at https://www.ebi.ac.uk/biostudies/arrayexpress and Genome Sequence Archive for Human of the China National Center for Bioinformation at https://ngdc.cncb.ac.cn/gsa-human and can be accessed with E‐MTAB‐6701 and HRA005470.

## References

[1] Yang H , Rao Z . Structural biology of SARS‐CoV‐2 and implications for therapeutic development. Nat Rev Microbiol. 2021;19:685‐700. doi:10.1038/s41579-021-00630-8 34535791 PMC8447893

[2] Rosenbloom JI , Raghuraman N , Carter EB , Kelly JC . Coronavirus disease 2019 infection and hypertensive disorders of pregnancy. Am J Obstet Gynecol. 2021;224:623‐624. doi:10.1016/j.ajog.2021.03.001 33675794 PMC7926183

[3] Wei SQ , Bilodeau‐Bertrand M , Liu S , Auger N . The impact of COVID‐19 on pregnancy outcomes: a systematic review and meta‐analysis. CMAJ. 2021;193:E540‐E548. doi:10.1503/cmaj.202604 33741725 PMC8084555

[4] Boettcher LB , Metz TD . Maternal and neonatal outcomes following SARS‐CoV‐2 infection. Semin Fetal Neonatal Med. 2023;28:101428. doi:10.1016/j.siny.2023.101428 37105860 PMC10005973

[5] Vento‐Tormo R , Efremova M , Botting RA , et al. Single‐cell reconstruction of the early maternal–fetal interface in humans. Nature. 2018;563:347‐353. doi:10.1038/s41586-018-0698-6 30429548 PMC7612850

[6] Greenbaum S , Averbukh I , Soon E , et al. A spatially resolved timeline of the human maternal–fetal interface. Nature. 2023;619:595‐605. doi:10.1038/s41586-023-06298-9 37468587 PMC10356615

[7] Mor G , Aldo P , Alvero AB . The unique immunological and microbial aspects of pregnancy. Nat Rev Immunol. 2017;17:469‐482. doi:10.1038/nri.2017.64 28627518

[8] James JL , Carter AM , Chamley LW . Human placentation from nidation to 5 weeks of gestation. Part I: what do we know about formative placental development following implantation? Placenta. 2012;33:327‐334. doi:10.1016/j.placenta.2012.01.020 22374510

[9] Patel J , Landers K , Mortimer RH , Richard K . Regulation of hypoxia inducible factors (HIF) in hypoxia and normoxia during placental development. Placenta. 2010;31:951‐957. doi:10.1016/j.placenta.2010.08.008 20869770

[10] Genbacev O , Zhou Y , Ludlow JW , Fisher SJ . Regulation of human placental development by oxygen tension. Science. 1997;277:1669‐1672. doi:10.1126/science.277.5332.1669 9287221

[11] Caniggia I , Mostachfi H , Winter J , et al. Hypoxia‐inducible factor‐1 mediates the biological effects of oxygen on human trophoblast differentiation through TGFβ3. J Clin Invest. 2000;105:577‐587. doi:10.1172/JCI8316 10712429 PMC289179

[12] Caniggia I , Winter JL . Adriana and Luisa Castellucci award lecture 2001 hypoxia inducible factor‐1: oxygen regulation of trophoblast differentiation in normal and pre‐eclamptic pregnancies—a review. Placenta. 2002;23:S47‐S57. doi:10.1053/plac.2002.0815 11978059

[13] Pringle KG , Kind KL , Sferruzzi‐Perri AN , Thompson JG , Roberts CT . Beyond oxygen: complex regulation and activity of hypoxia inducible factors in pregnancy. Hum Reprod Update. 2010;16:415‐431. doi:10.1093/humupd/dmp046 19926662 PMC2880912

[14] Burton GJ , Woods AW , Jauniaux E , Kingdom JCP . Rheological and physiological consequences of conversion of the maternal spiral arteries for uteroplacental blood flow during human pregnancy. Placenta. 2009;30:473‐482. doi:10.1016/j.placenta.2009.02.009 19375795 PMC2697319

[15] Khong TY , Robertson WB . Placenta creta and placenta praevia creta. Placenta. 1987;8:399‐409. doi:10.1016/0143-4004(87)90067-1 3684969

[16] Robertson WB , Khong TY , Brosens I , De Wolf F , Sheppard BL , Bonnar J . The placental bed biopsy: review from three European centers. Am J Obstet Gynecol. 1986;155:401‐412. doi:10.1016/0002-9378(86)90843-4 3526901

[17] Dommisse J , Tiltman AJ . Placental bed biopsies in placental abruption. BJOG. 1992;99:651‐654. doi:10.1111/j.1471-0528.1992.tb13848.x 1390469

[18] Smith GCS , Crossley JA , Aitken DA , et al. First‐trimester placentation and the risk of antepartum stillbirth. JAMA. 2004;292:2249‐2254. 15536112 10.1001/jama.292.18.2249

[19] Luo Z , Tian M , Yang G , et al. Hypoxia signaling in human health and diseases: implications and prospects for therapeutics. Sig Transduct Target Ther. 2022;7:218. doi:10.1038/s41392-022-01080-1 PMC9261907 35798726

[20] Bruick RK . Oxygen sensing in the hypoxic response pathway: regulation of the hypoxia‐inducible transcription factor. Genes Dev. 2003;17:2614‐2623. doi:10.1101/gad.1145503 14597660

[21] Lee P , Chandel NS , Simon MC . Cellular adaptation to hypoxia through hypoxia inducible factors and beyond. Nat Rev Mol Cell Biol. 2020;21:268‐283. doi:10.1038/s41580-020-0227-y 32144406 PMC7222024

[22] Xi C , Yan Z , Bai D , et al. Immune rebalancing at the maternal‐fetal interface of maternal SARS‐CoV‐2 infection during early pregnancy. Protein Cell. 2024;15:460‐473. doi:10.1093/procel/pwae006 38441496 PMC11131034

[23] Wei J , Liu X , Xiao W , et al. (2023). Phospholipid remodeling and its derivatives are associated with COVID‐19 severity. J Allergy Clin Immunol. 2023;151(5):1259–1268. doi:10.1016/j.jaci.2022.11.032 36736798 PMC9891787

[24] Zhou Y , Zhou B , Pache L , et al. Metascape provides a biologist‐oriented resource for the analysis of systems‐level datasets. Nat Commun. 2019;10:1523. doi:10.1038/s41467-019-09234-6 30944313 PMC6447622

[25] Liberzon A , Birger C , Thorvaldsdóttir H , Ghandi M , Mesirov JP , Tamayo P . The Molecular Signatures Database Hallmark Gene Set Collection. Cell Sys. 2015;1:417–425. doi:10.1016/j.cels.2015.12.004. PMC4707969 26771021

[26] Jin S , Guerrero‐Juarez CF , Zhang L , et al. Inference and analysis of cell‐cell communication using Cell Chat. Nat Commun. 2021;12:1088. doi:10.1038/s41467-021-21246-9 PMC7889871 33597522

[27] Ritchie HE , Oakes DJ , Kennedy D , Polson JW . Early gestational hypoxia and adverse developmental outcomes. Birth Defects Res. 2017;109:1358‐1376. doi:10.1002/bdr2.1136 29105381

[28] Serebrovska ZO , Chong EY , Serebrovska TV , Tumanovska LV , Xi L . Hypoxia, HIF‐1α, and COVID‐19: from pathogenic factors to potential therapeutic targets. Acta Pharmacol Sin. 2020;41:1539‐1546. doi:10.1038/s41401-020-00554-8 33110240 PMC7588589

[29] Ferreira LMR , Meissner TB , Tilburgs T , Strominger JL . HLA‐G: at the interface of maternal–fetal tolerance. Trends Immunol. 2017;38:272‐286. doi:10.1016/j.it.2017.01.009 28279591

[30] Wakeland AK , Soncin F , Moretto‐Zita M , et al. Hypoxia directs human extravillous trophoblast differentiation in a hypoxia‐inducible factor‐dependent manner. Am J Pathol. 2017;187:767‐780. doi:10.1016/j.ajpath.2016.11.018 28167044 PMC5397702

[31] Hermiston ML , Xu Z , Weiss A . CD45: a critical regulator of signaling thresholds in immune cells. Annu Rev Immunol. 2003;21:107‐137. doi:10.1146/annurev.immunol.21.120601.140946 12414720

[32] Midha AD , Zhou Y , Queliconi BB , et al. Organ‐specific fuel rewiring in acute and chronic hypoxia redistributes glucose and fatty acid metabolism. Cell Metab. 2023;35:504‐516.e5. doi:10.1016/j.cmet.2023.02.007 36889284 PMC10077660

[33] Jia H , Liu C , Li D , et al. Metabolomic analyses reveal new stage‐specific features of COVID‐19. Eur Respir J. 2022;59:2100284. doi:10.1183/13993003.00284-2021 34289974 PMC8311281

[34] Semenza GL . HIF‐1: upstream and downstream of cancer metabolism. Curr Opin Genet Dev. 2010;20:51‐56. doi:10.1016/j.gde.2009.10.009 19942427 PMC2822127

[35] Semenza GL . Hypoxia‐inducible factors in physiology and medicine. Cell. 2012;148:399‐408. doi:10.1016/j.cell.2012.01.021 22304911 PMC3437543

[36] Yamashita T , Honda M , Takatori H , et al. Activation of lipogenic pathway correlates with cell proliferation and poor prognosis in hepatocellular carcinoma. J Hepatol. 2009;50:100‐110. doi:10.1016/j.jhep.2008.07.036 19008011

[37] Zhang L , Fan Y , Yang Z , Yang M , Wong C‐Y . NIR‐II‐driven and glutathione depletion‐enhanced hypoxia‐irrelevant free radical nanogenerator for combined cancer therapy. J Nanobiotechnol. 2021;19:265. doi:10.1186/s12951-021-01003-2 PMC8420023 34488803

[38] Gupta GS . The lactate and the lactate dehydrogenase in inflammatory diseases and major risk factors in COVID‐19 patients. Inflammation. 2022;45:2091‐2123. doi:10.1007/s10753-022-01680-7 35588340 PMC9117991

[39] Di Guardo F , Di Grazia FM , Di Gregorio LM , et al. Poor maternal‐neonatal outcomes in pregnant patients with confirmed SARS‐Cov‐2 infection: analysis of 145 cases. Arch Gynecol Obstet. 2021;303:1483‐1488. doi:10.1007/s00404-020-05909-4 33389111 PMC7778712

[40] Nie X , Qian L , Sun R , et al. Multi‐organ proteomic landscape of COVID‐19 autopsies. Cell. 2021;184:775‐791.e14. doi:10.1016/j.cell.2021.01.004 33503446 PMC7794601

[41] Yuen K‐S , Ye Z‐W , Fung S‐Y , Chan C‐P , Jin D‐Y . SARS‐CoV‐2 and COVID‐19: the most important research questions. Cell Biosci. 2020;10:40. doi:10.1186/s13578-020-00404-4 32190290 PMC7074995

[42] Rutkai I , Mayer MG , Hellmers LM , et al. Neuropathology and virus in brain of SARS‐CoV‐2 infected non‐human primates. Nat Commun. 2022;13:1745. doi:10.1038/s41467-022-29440-z 35365631 PMC8975902

[43] Jauniaux E , Watson AL , Hempstock J , Bao Y‐P , Skepper JN , Burton GJ . Onset of maternal arterial blood flow and placental oxidative stress. Am J Pathol. 2000;157:2111‐2122. doi:10.1016/S0002-9440(10)64849-3 11106583 PMC1885754

[44] Valencia DN . Brief review on COVID‐19: the 2020 pandemic caused by SARS‐CoV‐2. Cureus. 2020;12:e7386. doi:10.7759/cureus.7386 32337113 PMC7179986

[45] Yockey LJ , Lucas C , Iwasaki A . Contributions of maternal and fetal antiviral immunity in congenital disease. Science. 2020;368:608‐612. doi:10.1126/science.aaz1960 32381717

