# Supplementary material for: Maternal infection with SARS‐CoV‐2 during early pregnancy induces hypoxia at the maternal–fetal interface
Source: Cell Prolif. 2024 Oct 7;58(2):e13749. doi: 10.1111/cpr.13749 (PMC11839197; doi:10.1111/cpr.13749)
Supplement: Supplementary file 1 — Data S1. Supporting Information. [file CPR-58-e13749-s002.docx]

**Supplementary materials**


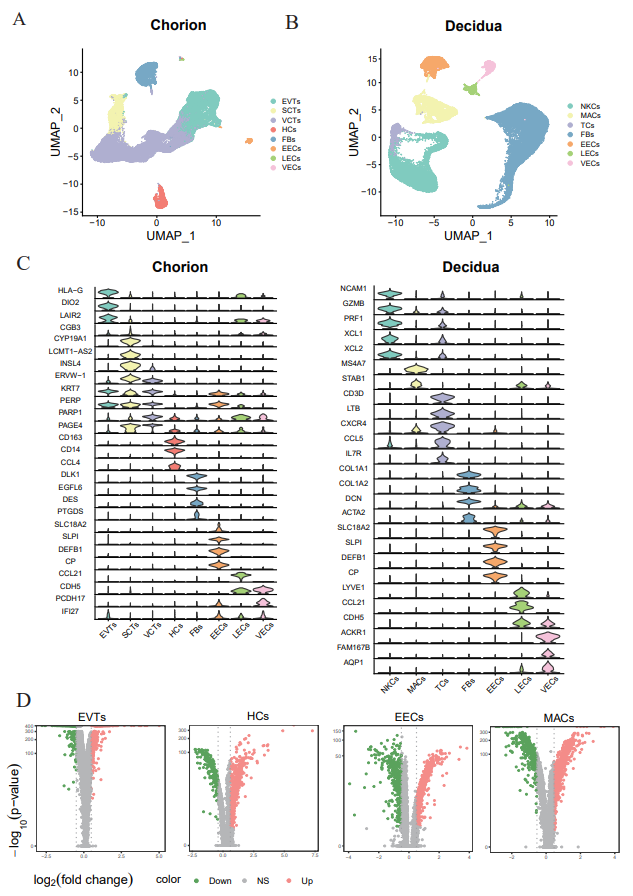


**Supplemental Figure 1 (Figure S1)**

(A) UMAP plots of 6-8 weeks placental chorion tissues colored by cell type. (B) UMAP plots of 6-8 weeks maternal decidua tissues colored by cell type. (C) Violin plots of key gene expression markers used for annotations of cells from 6-8 weeks placental chorion and maternal decidua tissues. (D) Volcano plot showing the DEGs in immune cells include HCs, MACs and Trophoblast cells include EVTs, EECs of placental chorion and maternal decidua at 6-8 weeks from pregnant women infected with SARS-CoV-2.


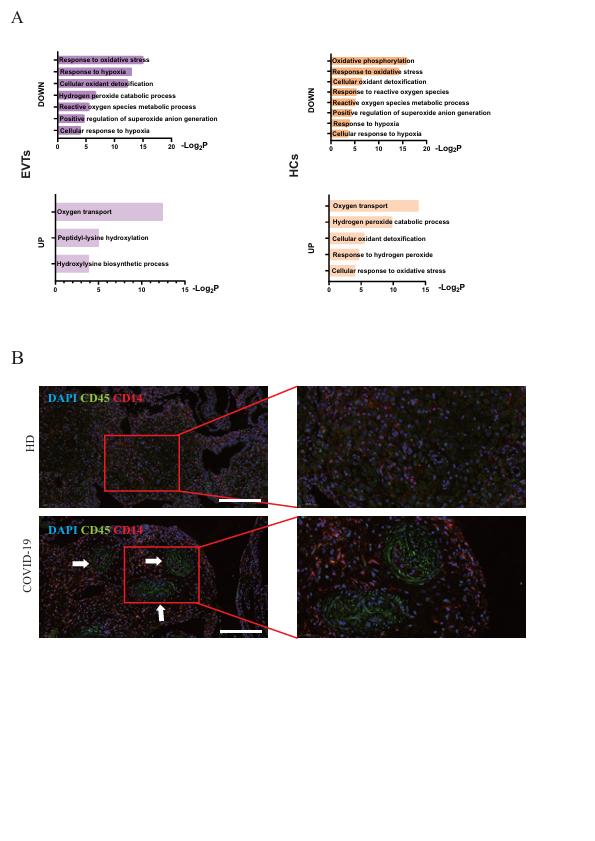


**Supplemental Figure 2 (Figure S2)**

(A) Gene Ontology (GO) enrichment analysis of upregulated and downregulated DEGs in EVTs and HCs of maternal decidua tissues from pregnant women infected with SARS-CoV-2. (B) Immunofluorescence staining of CD45 and CD14 in maternal decidua of the 6-8 weeks COVID-19 group. Scale bar = 200μm.

**Supplemental Table 1 (Table S1). Maternal clinical characteristics**

|  | **Chorion**  **of the 1st trimester (HD)** | **Chorion**  **of the 1st trimester (COVID-19)** | **Decidua**  **of the 1st**  **trimester (HD)** | **Decidua**  **of the 1st**  **trimester (COVID-19)** | **Placenta**  **of the 2nd**  **trimester (HD)** | **Placenta**  **of the 2nd**  **trimester (COVID-19)** |
| --- | --- | --- | --- | --- | --- | --- |
| **Maternal Age**  **(mean)** | 26-32  （27.5） | 26-34  （28.2） | 28-35  (29.2) | 26-33  (26.75) | 32-36  (34) | 26-38  (32) |
| **Gestational age at elective termination**  **(week)** | 6-8W | 6-8W | 6-8W | 6-8W | 12-16W | 12-16W |
| **Days of gestation by LMP (day)** | 53.8±5.4 | 55.4 ± 5.1 | 55.2±5.8 | 54.5 ± 5.3 | 100.5 ± 6.7 | 95.7 ± 10.7 |
| **Systolic blood pressure, mmHg**  **(mean)** | 85-103  (97.2) | 87-108  (99.4) | 89-110  (101.6) | 94-108  (102.5) | 86-141  (107) | 95-122  (110.5) |
| **Diastolic blood pressure, mmHg**  **(mean)** | 52-73  (62.4) | 53-71  (65.6) | 63-72  (67.5) | 64-71  (68.75) | 64-75  (69.5) | 69-82  (74.3) |
| **Maternal chronic health conditions** | no | no | no | no | no | no |

**Supplemental Table 2 (Table S2).**

**Details of clinical characteristics of subjects included in the study**

| **Group** | **Subject ID** | **Gestational age at elective termination(weeks)** | **SARS-CoV-2 infection** | **Fever** | **Maximum fever temperature (℃)** | **Gestational age at SARS-CoV-2 infection(weeks)** |
| --- | --- | --- | --- | --- | --- | --- |
|  | HD1 | 6 | no | --- | --- | --- |
|  | HD2 | 6 | no | --- | --- | --- |
|  | HD3 | 7 | no | --- | --- | --- |
|  | HD4 | 8 | no | --- | --- | --- |
|  | HD5 | 7 | no | --- | --- | --- |
| **HD** | HD6 | 12 | no | --- | --- | --- |
| **(Healthy** | HD7 | 13 | no | --- | --- | --- |
| **Donor)** | HD8 | 12 | no | --- | --- | --- |
|  | HD9 | 16 | no | --- | --- | --- |
|  | HD10 | 13 | no | --- | --- | --- |
|  | HD11 | 13 | no | --- | --- | --- |
|  | HD12 | 14 | no | --- | --- | --- |
|  | HD13 | 16 | no | --- | --- | --- |
|  | COV1 | 8 | yes | no | --- | 2 |
|  | COV2 | 8 | yes | yes | 38 | 3 |
|  | COV3 | 7 | yes | yes | 38.5 | 1 |
|  | COV4 | 6 | yes | yes | 39 | 1 |
|  | COV5 | 7 | yes | yes | 38.7 | 2 |
|  | COV6 | 6 | yes | no | --- | 3 |
|  | COV7 | 6 | yes | yes | 37.8 | 1 |
|  | COV8 | 8 | yes | yes | 38 | 2 |
|  | COV9 | 6 | yes | yes | 38 | 1 |
| **COVID-** | COV10 | 7 | yes | yes | 38.5 | 3 |
| **19** | COV11 | 12 | yes | no | --- | 2 |
|  | COV12 | 15 | yes | yes | 38.5 | 3 |
|  | COV13 | 12 | yes | yes | 39.2 | 2 |
|  | COV14 | 13 | yes | yes | 39 | 2 |
|  | COV15 | 12 | yes | yes | 38.7 | 1 |
|  | COV16 | 15 | yes | yes | 38.5 | 1 |
|  | COV17 | 13 | yes | no | --- | 3 |
|  | COV18 | 15 | yes | yes | 37.8 | 2 |
|  | COV19 | 12 | yes | yes | 39 | 1 |
|  | COV20 | 15 | yes | yes | 38 | 1 |
|  | COV21 | 14 | yes | yes | 39.3 | 2 |
